# Genome Sequence of *Riemerella anatipestifer* Strain RCAD0122, a Multidrug-Resistant Isolate from Ducks

**DOI:** 10.1128/genomeA.00332-16

**Published:** 2016-05-05

**Authors:** Xiao-Heng Song, Wang-Shu Zhou, Jiang-Bo Wang, Ma-Feng Liu, Ming-Shu Wang, An-Chun Cheng, Ren-Yong Jia, Shun Chen, Kun-Feng Sun, Qiao Yang, Ying Wu, Xiao-Yue Chen, De-Kang Zhu

**Affiliations:** a Research Center of Avian Diseases, College of Veterinary Medicine of Sichuan Agricultural University, Chengdu, Sichuan, China; b Key Laboratory of Animal Disease and Human Health of Sichuan Province, Chengdu, Sichuan, China; c Institute of Preventive Veterinary Medicine of Sichuan Agricultural University, Chengdu, Sichuan, China

## Abstract

*Riemerella anatipestifer* is an important pathogenic bacterium in waterfowl and other avian species. We present here the genome sequence of *R. anatipestifer* RCAD0122, a multidrug-resistant strain isolated from infected ducks. The isolate contains at least nine types of antibiotic resistance-associated genes.

## GENOME ANNOUNCEMENT

*Riemerella anatipestifer* is a Gram-negative, non-spore-forming, and rod-shaped bacterium, which can infect domestic ducks, turkeys, and various other birds (1). To date, only one genome sequence of multidrug-resistant *R. anatipestifer* strains has been reported (2). RCAD0122, a strain isolated from the livers of ducks in China, has been proven to be resistant to multiple antibiotics; these antibiotics include β-lactamases, fluoroquinolones, chloramphenicol, lincosamide, sulfonamides, aminoglycosides, tetracyclines, glycopeptide antibiotics, and erythrocin. Because of this, the genome of RCAD0122 was sequenced. We think the genome sequence of RCAD0122 may facilitate a better understanding of the resistance mechanism of *R. anatipestifer* compared to that of the sequenced resistance strains (2, 3). *R. anatipestifer* has a very wide drug resistance spectrum and is resistant to many of the antibiotics mentioned in a previous report (4).

The strain RCAD0122 was sequenced using Illumina HiSeq 2500. *De novo* assembly was performed using Velvet version 1.2.09 (5). The genome was annotated by the NCBI Prokaryotic Genome Annotation Pipeline (PGAP) (http://www.ncbi.nlm.nih.gov/genome/annotation_prok). The potential of each connection between the scaffolds was analyzed. Annotated features include putative coding sequences (CDSs), coding genes, rRNAs, tRNAs, and noncoding RNAs (ncRNAs). Resistance gene identification was performed using the Comprehensive Antibiotic Resistance Database (6). The results indicated that the genome has 2,021 genes, 2,021 CDSs, 36 tRNAs, 6 rRNAs, and 3 ncRNAs. The sequencing depth was 1 Gb, the total length was 2,202,920 bp, the G+C content was 35.01%, and the genome coverage was 78.64%.

To date, four families of active efflux systems have been elucidated (7, 8). In one previous study, the small multidrug resistance protein (SMR) family and major facilitator superfamily (MFS) were found in ATCC 11845 (2). MFS, a tetracycline resistance efflux pump; *lasE*, conferring resistance to lincosamide (9); *MdIB*, which is an ABC-type multidrug transport system; and AcrAB-TolC, which is related to fluoroquinolone, chloramphenicol, β-lactam, and rifampin resistance, were found in RCAD0122 (10, 11). Nine types of resistance-associated genes were found in the strain RCAD0122: *aph*, an aminoglycoside resistance gene; *dhfR*, which confers resistance to trimethoprim; *folP* and *folB*, which are sulfonamide resistance genes; *parE*, the fluoroquinolone resistance gene; *vanC*, a vancomycin resistance gene; TLA-2, a class A β-lactamase; OXA-209, a class D β-lactamase (12); *cat,* a chloramphenicol acetyltransferase resistance gene; *tet*(X), a tetracycline inactivation gene; and e*reB*, a macrolide esterase gene (13). RCAD0122 was found to have many drug resistance genes and multidrug efflux pumps, which is probably why it has such a wide range of drug resistance; in addition, the results showed RCAD0122 to have transposase and integrase genes, which can explain the existence of so many resistance genes in this strain.

Overall, the genome sequence of RCAD0122 can provide a genetic background for understanding the resistance mechanism of *R. anatipestifer* using comparative genomics.

### Nucleotide sequence accession numbers.

This whole-genome shotgun project has been deposited in DDBJ/ENA/GenBank under the accession no. LUDU00000000. The version described in this paper is the first version, LUDU01000000.
